# RNAi-mediated gene silencing of *Phlebotomus papatasi* defensins favors *Leishmania major* infection

**DOI:** 10.3389/fphys.2023.1182141

**Published:** 2023-05-09

**Authors:** Barbora Vomáčková Kykalová, Fabiana Sassù, Petr Volf, Erich Loza Telleria

**Affiliations:** Department of Parasitology, Faculty of Science, Charles University, Prague, Czechia

**Keywords:** sand fly, innate immunity, relish, antimicrobial peptides, knockdown, leishmania, defensin

## Abstract

**Introduction:** Production of different antimicrobial peptides (AMPs) is one of the insect’s prominent defense strategies, regulated mainly by Toll and immune deficiency (IMD) humoral pathways. Here we focused mainly on two AMPs of *Phlebotomus papatasi*, vector of *Leishmania major* parasites, their association with the relish transcription factor and the effective participation on *Leishmania* infection.

**Methods and results:** We further characterized the role of previously described gut-specific *P. papatasi* defensin (PpDef1) and identified the second defensin (PpDef2) expressed in various sand fly tissues. Using the RNAi-mediated gene silencing, we report that the silencing of *PpDef1* gene or simultaneous silencing of both defensin genes (*PpDef1* and *PpDef2*) resulted in increased parasite levels in the sand fly (detectable by PCR) and higher sand fly mortality. In addition, we knocked down relish, the sole transcription factor of the IMD pathway, to evaluate the association of the IMD pathway with AMPs expression in *P. papatasi*. We demonstrated that the relish gene knockdown reduced the expression of *PpDef2* and *attacin*, another AMP abundantly expressed in the sand fly body.

**Conclusions:** Altogether, our experiments show the importance of defensins in the sand fly response toward *L. major* and the role of the IMD pathway in regulating AMPs in *P. papatasi*.

## 1 Introduction

Antimicrobial peptides (AMPs), including defensins, are prominent effector molecules of innate insect immunity. Their transcription is regulated mainly by two humoral pathways, Toll and immune deficiency (IMD) (Hoffmann, 2004). Very briefly, pathogens are recognized by transmembrane receptors, which leads to numerous signaling events. The signaling cascade terminates by translocation of transcriptional factors dorsal and relish belonging to Toll and IMD pathways, respectively, into the cell nucleus followed by AMPs transcription (De Gregorio et al., 2002).

In insect vectors, the IMD pathway has a role in the innate immune response against parasites. For example, the over-activation of IMD-mediated response in three anopheline mosquitoes caused the reduction of *Plasmodium falciparum* infection (Garver et al., 2009). Similarly, this increased response in the sand fly *Lutzomyia longipalpis* caused the reduction of *Leishmania* parasites (Telleria et al., 2012). The IMD-mediated response is controlled by the nuclear factor-kappa B (NF-κB) protein sub-family members, also known as relish proteins in arthropods (Dushay et al., 1996; Huguet et al., 1997). The importance of relish in controlling the response against parasites was shown when the knockout of relish in the sand fly *Phlebotomus papatasi* caused increased numbers of *Leishmania major* parasites and bacteria loads in the sand fly gut (Louradour et al., 2019).

Insects have a broad repertoire of AMP molecules acting in synergy to effectively control a plethora of infectious agents, although they are often targeted for specific microorganisms (Bulet et al., 1999). In the present work, we focused on defensins, small 4-kDa peptides with 6 cysteines and 3 intramolecular disulfide bridges (Cociancich et al., 1993). Insect defensins act against a wide spectrum of Gram-positive (G+) and Gram-negative (G-) bacteria and fungi. Their mechanisms of action involve membrane perforation, blocking the ion channel formation, or targeting specific pathogen structures (Bulet et al., 1999). Defensins, however, have anti-protozoan activity as well; for example, purified defensins from the blow fly *Phormia terranovae* and dragonfly *Aeschna cyanea* acted against *Plasmodium gallinaceum* by reducing the oocysts number in mosquitoes and altering sporozoite morphology (Shahabuddin et al., 1998). In addition, a recombinant defensin produced from *Triatoma pallidipennis* showed *in vitro* lytic activity on *Trypanosoma* and *Leishmania* parasites (Díaz-Garrido et al., 2021).

We are interested in the *P. papatasi* study model because it stands out as a main vector of *L. major* parasites, causing cutaneous leishmaniasis and affecting hundreds of thousands of human lives yearly (Akhoundi et al., 2016). Understanding how the sand fly reacts to *Leishmania* infection may reveal alternative targets for transmission control strategies. Several studies have been developed in this direction, but many aspects of this multifactor relationship remain uncovered (Telleria et al., 2018). In *Phlebotomus duboscqi*, another vector of *L. major*, a recombinant defensin showed *in vitro* antiparasitic activity against *Leishmania* promastigotes (Boulanger et al., 2004). In *L. longipalpis,* a vector of *L. infantum* in the Americas, the gene expression of AMPs defensin2 (LlDef2), attacin (LlAtt), and cecropin (LlCec) was increased during *Leishmania infantum* infection, and the systemic silencing of *LlDef2* gene resulted in a slight change in *L. infantum* detection (Telleria et al., 2021b). Although the development of *Leishmania* parasites in the sand fly is restricted to the gut, the parasitic infection seems to trigger a systemic response produced in fat body cells.

Interestingly, recent results of our team also reveal a tissue-specific response in sand flies: the gene expression of a gut-specific *P. papatasi* defensin (PpDef1) was increased after *L. major* infection in bacteria-depleted sand flies (Kykalová et al., 2021). However, it is unknown whether the IMD pathway regulates this defensin and if PpDef1has any role in controlling the parasites in the sand fly gut. To address these questions, we silenced relish by RNAi-mediated gene silencing and followed the AMPs expression by qPCR. We investigated these immunity gene expressions in dissected guts or carcasses. The Toll pathway can also regulate AMPs expression (Hoffmann, 2004; Tinoco-Nunes et al., 2016). Nevertheless, we did not address it in the present study. We also silenced two defensin genes in bacteria-depleted sand flies to evaluate the effect on *L. major* infection.

## 2 Materials and methods

### 2.1 Sand flies and antibiotic treatment

*Phlebotomus papatasi* colony was established from field-caught sand flies from Turkey in 2005. Sand flies were kept under standard conditions at 26°C, 45% relative humidity, and 14 h light/10 h dark photoperiod (Volf and Volfova, 2011). Adult sand flies were fed on a 30% sucrose solution offered on cotton wool. For experimental infections, adult females were fed on a 30% sucrose solution containing an antibiotic cocktail (AtbC) to deplete gut bacteria. AtbC was adapted from (Kelly et al., 2017): 100 units/mL of penicillin (BB Pharma, Martin, Slovakia), 50 μg/mL of gentamicin (Sandoz, Boucherville, Canada), and 4 μg/mL of clindamycin (Sigma–Aldrich, Saint Louis, MI, United States). The bacteria depleted sand flies were used to access the immune response caused mainly by *L. major*. The efficiency of our choice of AtbC and a possible interference with parasite development in the vector was previously addressed in this parasite-vector pair by our team, and no negative correlation between parasite development and AtbC was detected (Kykalová et al., 2021).

### 2.2 Parasites and experimental infections of sand flies

*Leishmania major* parasites (FV1 MHOH/IL/80/Friedlin) were cultivated in Medium199 (Sigma–Aldrich) at 23°C, supplemented with 10% heat-inactivated fetal bovine serum (Thermo Fisher Scientific, Carlsbad, CA, United States), 1% BME vitamins (Sigma–Aldrich), 2% of sterile urine, and 250 μg/mL amikacin (Medopharm, Pozorice, Czech Republic). Adult females had access to AtbC in sucrose solution served *ad libitum* and changed daily for 5 days after eclosion, during the experimental infection and after infection. On day 5, females were fed through chicken skin membrane on defibrinated sheep blood (LabMediaServis, Jaromer, Czech Republic) with AtbC, seeded with 10^6^ *L. major* promastigotes/mL. Blood-fed females were separated, kept under the same conditions described above, and dissected at different time intervals (indicated in figure legends). Guts (without Malpighian tubules) and carcasses (i.e., all other tissues) were dissected in sterile saline solution, collected in pools of 10, and stored at −80°C until processing.

### 2.3 *P. papatasi* AMPs and relish gene sequences

*Phlebotomus papatasi* relish (PpRel) (PPAI012820), attacin (PpAtt) (PPAI003791), and PpDef1 (PPAI004256) gene sequences were previously identified (Louradour et al., 2019; Kykalová et al., 2021; Sloan et al., 2021) and are available from the Vector Base website (Amos et al., 2022). A second *P. papatasi* defensin sequence (PpDef2) was identified by similarity using the *L. longipalpis* defensin sequences (Telleria et al., 2021b) as a query to search on the *P. papatasi* RNAseq database publicly available from the Vector Base using blast search tools. The PpDef2 was amplified by PCR (Table 1) and *P. papatasi* cDNA template and sequenced for confirmation.

**TABLE 1:** Oligonucleotides.

Gene name	Reference	Sequence
**Actin (PpAct)**	Kykalová et al. (2021)	5′ GCA​CAT​CCC​TGG​AGA​AAT​CCT​AT 3′
(PPAI004850)		5′ GGA​AAG​ATG​GCT​GGA​AGA​GAG​AT 3′
**Ribosomal protein L8 (PpRibL8)**	Kykalová et al. (2021)	5′ GAC​ATG​GAT​ACC​TCA​AGG​GAG​TC 3′
(PPAI008202)		5′ TTG​CGG​ATC​TTA​TAG​CGA​TAG​GG 3′
**Relish (PpRel)**	Louradour et al. (2019)	5′ GGA​GCT​TCC​GTT​CCC​ATC​AA 3′
(PPAI012820)		5′ TCG​TCC​TCT​CGA​ATA​GCC​CA 3′
**Attacin (PpAtt)**	Kykalová et al. (2021)	5′ GCC​ATT​TCT​GCT​GCG​TAC​TC 3′
(PPAI003791)		5′ GAG​GCA​CCA​AGT​ACA​CGA​CA 3′
**Defensin1 (PpDef1)**	Kykalová et al. (2021)	5′ GCC​CGG​TTA​AAG​ACG​ATG​TAA​AG 3′
(PPAI004256)		5′ AGT​TGG​TCC​AAG​GAT​ATC​GCA​AG 3′
**Defensin2 (PpDef2)**	present study	5′ ATT​CAC​GCC​AAA​AAC​GAG​CC 3′
(PPAI010650)		5′ CGA​TAC​AAT​GGG​CAG​CAC​AAG 3′
**PpDef2 confirmation**	present study	5′ TGC​GTA​CGT​TCT​TGG​TAG​TAG​T 3′
(PPAI010650)		5′ TGTGCAGACAGCCTTTGA 3′
***Leishmania* actin**	Di-Blasi et al. (2015)	5′ GTC​GTC​GAT​AAA​GCC​GAA​GGT​GGT​T 3′
		5′ TTG​GGC​CAG​ACT​CGT​CGT​ACT​CGC​T 3′
**dsRel (PpRel)**	present study	5′ TAA​TAC​GAC​TCA​CTA​TAG​GGA​GAACT​CTT​CTG​ACA​TTC​CCC​TGA​C 3′
		5′ TAA​TAC​GAC​TCA​CTA​TAG​GGA​GATTT​GAT​GGG​AAC​GGA​AGC​TCC​C 3′
**dsDef1 (PpDef1)**	present study	5′ TAA​TAC​GAC​TCA​CTA​TAG​GGA​GATGT​AAA​GGA​CCC​TGT​GGA​GGA 3′
		5′ TAA​TAC​GAC​TCA​CTA​TAG​GGA​GAATC​ATC​GCA​CCA​TCC​TCC​TG 3′
**dsDef2 (PpDef2)**	present study	5′ TAA​TAC​GAC​TCA​CTA​TAG​GGA​GACTT​GTC​GTT​GTT​GTG​GGA​G 3′
		5′ TAA​TAC​GAC​TCA​CTA​TAG​GGA​GAAAG​CAG​CAT​GAC​CAA​CTC 3′
**dsLacZ (LacZ)**	(adapted from Molina-Cruz et al., (2008)	5′ TAA​TAC​GAC​TCA​CTA​TAG​GGA​GATAT​CCG​CTC​ACA​ATT​CCA​CA 3′
		5′ TAA​TAC​GAC​TCA​CTA​TAG​GGA​GAGAG​TCA​GTG​AGC​GAG​GAA​GC 3′

Accession numbers of gene sequences obtained from VectorBase database are indicated below gene names.

*Underlined nucleotides indicate T7 Polymerase Promoter.

Bold characters indicate gene name followed by gene abbreviation.

The conserved domain present in the PpDef2 amino acid sequence was identified using the InterPro (Blum et al., 2021) and the NCBI Conserved Domain Database (Lu et al., 2020) tools to support its identification. Similarities between the PpDef2 and other insect defensins were assessed by the MUSCLE multiple sequence alignment tool (Edgar, 2004) built-in Geneious 7.1.9 software (Biomatters, Auckland, New Zealand). A phylogram analysis was performed using the Maximum Likelihood method and Whelan and Goldman model (Whelan and Goldman, 2001), allowing for evolutionarily invariable sites (WAG + I) with a bootstrap value of 400 repetitions in MEGA X 10.0.5 software (Kumar et al., 2018).

### 2.4 dsRNA synthesis and microinjections in the sand flies

Templates for dsRNA synthesis were amplified by PCR using *P. papatasi* cDNA and gene-specific primers for PpRel, PpDef1, and PpDef2 containing the T7 promoter sequence on the 5’ends (Table 1). The template for control dsRNA was amplified from p-GEM-T Easy plasmid (Promega, Madison, WI, United States) using dsLacZ primers (Table 1). The PCR cycling conditions were as follows: 95°C for 3°min; 34 amplification cycles (95°C for 30°s; 60°C for 30°s, 72°C for 1 min); and 72°C for 5 min. Amplicons were visualized on 1% agarose gel and purified using E.Z.N.A. Gel Extraction kit (Omega Bio-tek, Norcross, GA, United States). Purified templates were used in dsRNA synthesis using the MEGAscript RNAi Kit (Invitrogen, Carlsbad, CA, United States) according to the manufacturer’s instructions. Gene-specific dsRNA were lyophilized and resuspended in sterile H_2_O to a final concentration of 4.5 μg/μL.

For gene silencing, dsRNA was manually microinjected using Nanoject II microinjector (Drummond, Broomall, PA, United States) into the thorax of adult sand fly females anesthetized on ice (Sant’Anna et al., 2008). To test the efficiency of gene silencing, 1–2°days old sucrose-fed colony females were microinjected with 32 nL (150 ng) of dsRNA specific for *PpRel* (dsRel), *PpDef1* (dsDef1), *PpDef2* (dsDef2) genes, and a non-related LacZ dsRNA (dsLacZ) as a control. After confirming the efficient gene silencing, that in our hands is usually achieved between first and third days post dsRNA injection, the dsRNA is used in further experimental conditions. Preliminary experiments showed that sugar and blood feedings do not interfere with gene silencing by RNAi pathway.

In addition, to study the role of defensins during *L. major* infection, 3-day infected females were separated into three groups. The first experimental group was injected with 64 nL (300 ng) of dsDef1 to silence the gut-specific defensin. The second group was injected with a mixture of 32 nL (150 ng) of dsDef1 plus 32 nL (150 ng) of dsDef2 (dsDef1+2) to create an additive effect of silencing another defensin that was systemically expressed. The third group (control) was injected with 64 nL (300 ng) of dsLacZ. The choice of injecting dsRNA in 3-day infected females was based on two factors. First, to match the period of efficient gene silencing with the time when PpDef1 is differentially expressed in the sand fly gut (72 h or 144 h post infection) (Kykalová et al., 2021). Second, to match with the crucial moment in *Leishmania* cycle in *P. papatasi* when blood digestion ends, and the parasites migrate to the anterior part of the sand fly gut (between 72°h and 96 h.post infection) (Dillon and Lane, 1993; Pruzinova et al., 2015).

### 2.5 RNA extraction and cDNA synthesis

Total RNA was extracted from the samples stored at −80°C using E.Z.N.A. Total RNA Kit I (Omega Bio-tek) following the manufacturer’s instructions. A DNA digestion step with RNase-free DNase I (Thermo Fisher Scientific, Waltham, MA, United States) was included to clean RNA from possible DNA residues. DNA-free RNA templates were used in a cDNA synthesis reaction with anchored-oligo (dT)_18_ primer using a Transcriptor First Strand cDNA Synthesis Kit (Roche Life Science, Rotkreuz, Switzerland) following the manufacturer’s instructions.

PCR amplification of *P. papatasi* actin (Table 1) was carried out to control cDNA synthesis using the same cycling conditions mentioned above. Amplicons were visualized on 1% agarose gel.

### 2.6 Relative gene-expression analysis

Quantitative PCR (qPCR) was prepared with cDNA samples, gene-specific primers (Table 1), and SYBR Green PCR Master mix (Roche) to detect the expressions of investigated genes using a LightCycler 480 thermocycler (Roche). Relative gene expression was calculated relative to *P. papatasi* endogenous control genes *actin* (*PpAct*) (PPAI004850) and *60S ribosomal protein subunit L8* (*PpRibL8*) (PPAI008202) using the following cycling conditions: 95°C for 10 min enzyme activation, 45 amplification cycles (95°C for 10°s, 60°C for 20°s; 72°C for 45 s) (Kykalová et al., 2021). Expression levels were expressed as the fold change compared to the control groups.

### 2.7 *L. major* development in *P. papatasi*

Sand fly guts were examined 6 days post parasite infection under a light microscope to determine the parasite loads and localization. Day 6 of infection represents a late stage when the defecation process is finished, and parasites start to migrate to the thoracic midgut and the stomodeal valve (Dostálová and Volf, 2012). In addition, this time point corresponds with the third day post dsRNA injection, when the gene silencing effects can still be detected (Sant’Anna et al., 2008; Sant’Anna et al., 2009; Coutinho-Abreu et al., 2010b; Telleria et al., 2012; Di-Blasi et al., 2019). A total of 65 females per group (dsDef1, dsDef1+2, and dsLacZ) from three independent experiments were dissected in sterile saline solution (NaCl 0.9%) and inspected under a ×40 magnification objective lens. Parasite loads in the gut were classified as light (below 100 parasites per gut), medium (between 100 and 1,000 parasites per gut), and heavy (above 1,000 parasites per gut), as it was previously described (Myskova et al., 2008). Also, the localization of parasites was evaluated and recorded in the abdominal midgut, thoracic midgut, cardia, and stomodeal valve (Sádlová et al., 2010).

Smears of randomly selected gut samples were prepared on glass slides for assessing the parasite development stages. Smears of individual guts were fixed with methanol and Giemsa-stained. Images of fifty randomly selected parasites per slide, 200 parasites from each group, were captured under a ×100 magnification objective lens in an Olympus BX51 microscope (OLYMPUS, Tokyo, Japan). Parasite cell width, length, and flagellum were measured using the microscope scale plugin in ImageJ 1.52a software (Abràmoff et al., 2004). Parasite morphological forms were identified according to previously published criteria as procyclic promastigotes (body length < 14 µm and flagellar length ≤ body length), elongated nectomonads (body length ≥ 14 µm), metacyclic promastigotes (body length < 14 µm and flagellar length ≥ 2× body length), and leptomonads (short nectomonads = remaining parasites) (Sádlová et al., 2010).

### 2.8 Mortality rate

To evaluate the possible negative effect of defensins silencing after *L. major* infection, numbers of alive and dead sand flies from experimental (dsDef1 and dsDef1+dsDef2) and control (dsLacZ) groups were recorded during three consecutive days post intrathoracic microinjections, and mortality rate for each group was calculated. Each of the three independent experiments contained a minimum of 50 female sand flies per group.

### 2.9 Statistical analysis

Statistical analyses and graphs were performed using GraphPad Prism software (GraphPad Software, La Jolla, CA, United States). Student *t*-test was applied to calculate significant differences in gene expression levels in the gut compared to carcass samples obtained from a single time point. Ordinary two-way ANOVA was applied to calculate significant differences in gene expression levels at several time points in gut and carcass samples from experimental groups compared to a control group. Two-way ANOVA was also applied to calculate significant differences in mortality rates between experimental (dsDef1 and dsDef1+2) and control (dsLacZ) groups. Both student *t*-test and two-way ANOVA methods were applied with Holm-Sidak correction for multiple comparisons. For analyzing several infection parameters, contingency tables were created, and Chi-square was applied to test significant differences between experimental and control groups injected with dsRNA. Subsequently, Fisher’s test was used for each category between the experimental and control group. Additional details were included in figure legends.

## 3 Results

### 3.1 *P. papatasi* defensin2 and relish expression in the sand fly tissues

Previously, the PpDef1 (PPAI004256) was identified as a gut-specific defensin (Kykalová et al., 2021). We searched for other defensin-coding sequences in the VectorBase database and identified the PPAI010650 sequence, here named PpDef2. The translated amino acid sequence has 98 residues, including six cysteine residues characteristic of the defensin superfamily signature domain (Supplementary Figure S1A). The phylogenetic analysis showed that the PpDef2 amino acid sequence is closely related to *P. duboscqi* defensin (P83404.3) and *L. longipalpis* LlDef2 (AKU77027.1). On the other hand, the gut-specific PpDef1 and *L. longipalpis* defensin4 (MW269863.1) form a separate clade (Supplementary Figure S1B). The PpDef2 gene expression in *P. papatasi* guts was highly variable after the *L. major* infection. Although it was slightly increased at 24 h and reduced at 72 h post-infection, none of the analyzed time points showed significant differences compared to the non-infected control group (Supplementary Figure S1C).

**FIGURE 1:**
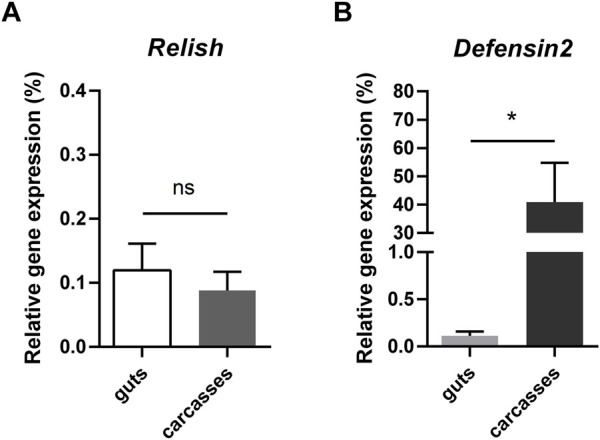
Expression of *P. papatasi* immunity genes in guts and carcasses. **(A, B)** Relative gene expression of *PpRel* and *PpDef2* genes guts and carcasses. The *y*-axis represents the relative expression as percentage compared to endogenous controls *PpAct* and *PpRibL8* genes. The *x*-axis indicates sand fly samples. Vertical bars represent the mean with standard error (SEM) of 3 biological replicates. Significant differences were calculated using Student *t*-test with Holm-Sidak’s correction (* *p* < 0.05).

We explored whether *PpRel* and *PpDef2* genes have gut-specific expressions. Both were expressed in dissected guts and carcasses of females from the colony (sucrose-fed). *PpRel* gene had similar expression levels when compared between guts and carcasses (Figure 1A). On the other hand, *PpDef2* gene was expressed significantly higher in carcasses than in guts (Figure 1B).

### 3.2 Relish correlation with AMPs expression

**FIGURE 2:**
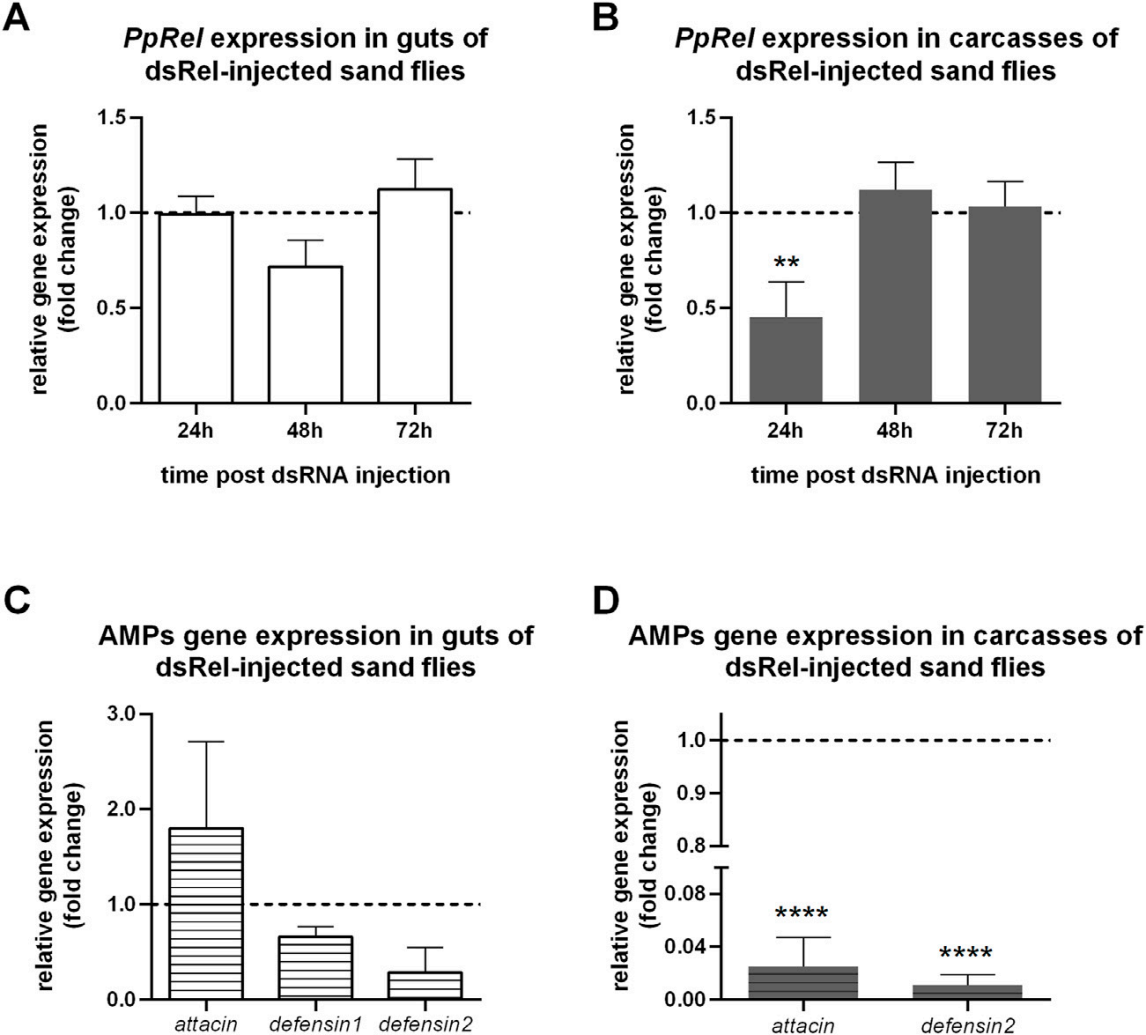
Expression of *P. papatasi* immunity genes after silencing of relish. **(A, B)** The relative expression of *PpRel* gene in dsRel-injected sand flies is represented in the *y*-axis, and time points when samples were collected post dsRNA injection are indicated in the *x*-axis. **(C, D)** The relative gene expression of AMPs at 24 h post dsRel injection is represented in the *y*-axis, and AMPs gene names are indicated in the *x*-axis. **(A, C)** White background color indicates guts. **(B, D)** Grey background color indicates carcasses samples. The relative expression was normalized to endogenous controls *PpAct* and *PpRibL8* genes and expressed as fold change compared to the dsLacZ control group collected at each correspondent time point (dotted line). Vertical bars represent the mean with standard error (SEM) of 3 biological replicates. Significant differences were calculated using two-way ANOVA with Holm-Sidak’s correction (**p < 0.01; ****p < 0.0001).

We hypothesized that the IMD pathway controlled the two defensins through relish transcription factor. To test this hypothesis, we injected 150 ng of PpRel dsRNA (dsRel) into sugar-fed *P. papatasi* females. This amount of dsRNA was previously used with good efficiency in sand flies, but no significant changes were observed in PpRel expression in guts (Figure 2A). On the other hand, a significant reduction occurred in carcasses at 24 h (Figure 2B). Then, we selected the sand fly samples that showed low PpRel expression collected at 24 h (carcass) and 48 h (gut) post dsRel injection to test if there was an alteration of expression in the AMPs genes. In sand flies injected with 150 ng of dsRel, there were no significant changes in AMPs gene expression in the selected gut samples (Figure 2C). At the same time, there was a significant reduction in the expression of *PpDef2* and *PpAtt* genes in the carcasses (Figure 2D).

### 3.3 Gene silencing of defensins and its effect on *Leishmania* infections

**FIGURE 3:**
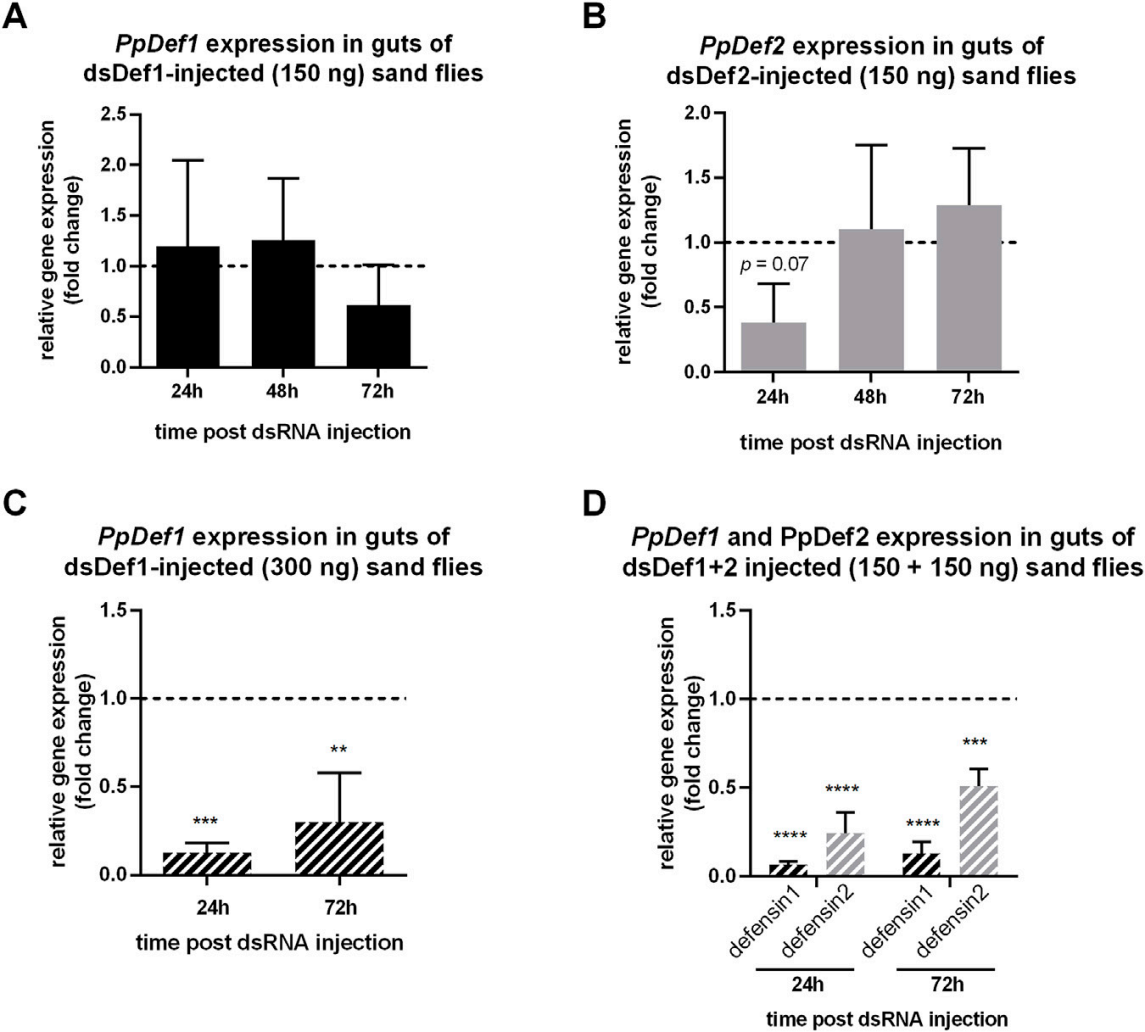
Gene expression of *P. papatasi* defensins genes after dsRNA injections. The relative expression of *PpDef1* (black) and *PpDef2* (light grey) genes in dsRNA-injected sand flies is represented in the *y*-axis. Time points when samples were collected post dsRNA injection are indicated in the *x*-axis. **(A, B)** Expression of *PpDef1* and *PpDef2* genes after 150 ng of gene-specific dsRNA in sugar-fed non-infected flies. **(C, D)** Expression of *PpDef1* and *PpDef2* genes in *Leishmania*-infected flies (white stripes) after 300 ng of dsDef1 or dsDef1+2 mixture. In infected sand flies, 24 h and 72 h post dsRNA injection correspond to day 4 and 6 post infection. The relative expression was normalized to endogenous control genes *PpAct* and *PpRibL8* and expressed as fold change compared to the dsLacZ control group collected at each correspondent time point (dotted line). Vertical bars represent the mean with standard error (SEM) of 3 biological replicates. Significant differences were calculated using two-way ANOVA with Holm-Sidak’s correction (**p < 0.01; ***p < 0.001; ****p < 0.0001).

In a first experimental setting, we injected a standard volume 32 nL (150 ng) of dsRNA intrathoracically into sugar-fed non-infected *P. papatasi* females to silence both defensins. While the injection of the dsDef1 did not result in successful gene silencing in the sand fly gut (Figure 3A), the injection of dsDef2 resulted in a reduced expression of *PpDef2* gene (Figure 3B) in the sand fly gut on the first day post-injection. For our control of dsRNA injection and gene silencing, we followed the *PpDef2* gene expression in carcasses and we detected a significant reduction on the first day post dsDef2 injection (Supplementary Figure S2). No significant changes were observed in *PpDef1* expression in sand fly guts after dsDef2 injection (Supplementary Figure S3A), nor in *PpDef2* after dsDef1 injection (Supplementary Figure S3B).

In a different experimental setting, on the third day post *Leishmania* infection, we increased the injected volume to double of dsDef1 (64 nL; 300 ng) in *P. papatasi* females. The higher volume of injected dsRNA resulted in similar survival rate as the standard volume. Significant silencing of *PpDef1* gene was observed on days 1 and 3 post-injection (Figure 3C). For comparison purposes, we tested the combined silencing of both *PpDef1* and *PpDef2* genes (considering the maximum volume that can be injected intrathoracically) by injecting 32 nL (150 ng) of each dsRNA into infected *P. papatasi* females. The silencing of both defensins in the sand fly gut was significant on days 1 and 3 post-injection (Figure 3D).

**FIGURE 4:**
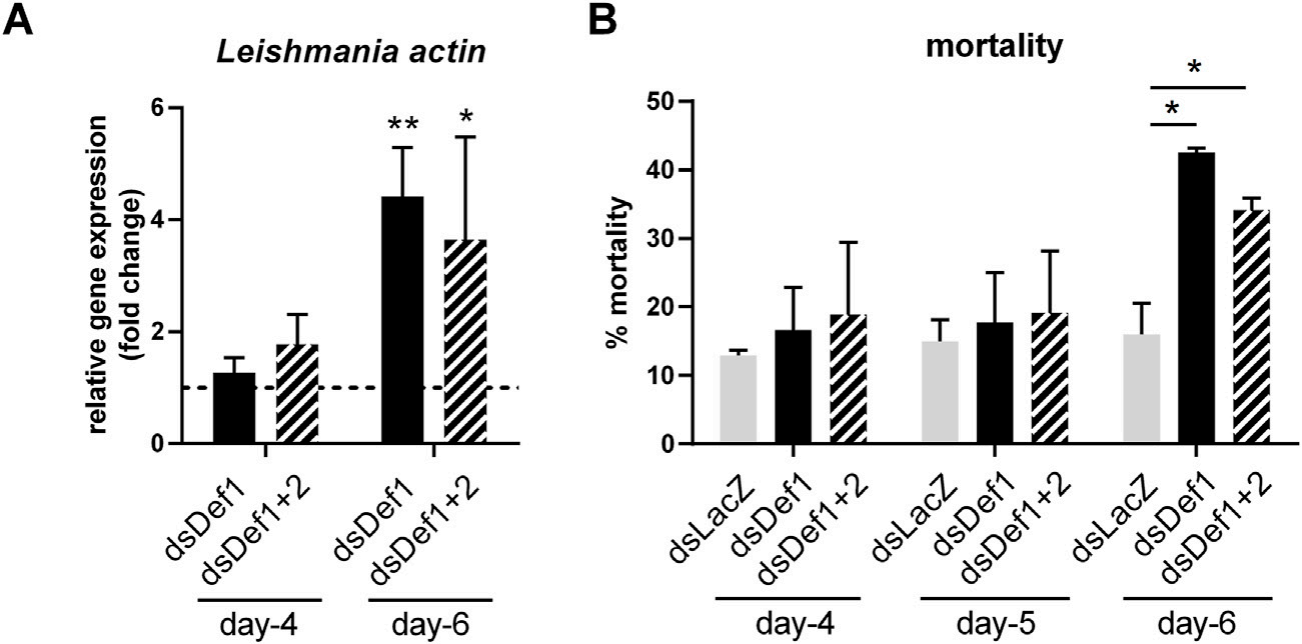
*Leishmania* actin expression and mortality after dsRNA injections in *Leishmania*-infected sand flies. **(A)** *Leishmania* actin expression in dsDef1 and dsDef1+2 injected groups are represented by black color and striped bars, respectively. The relative gene expression was normalized to endogenous control genes *PpAct* and *PpRibL8* and expressed as fold change compared to the dsLacZ control group collected at each correspondent time point (dotted line). **(B)** Mortality levels are expressed as a percentage in comparison to the total number of live sand flies (*y*-axis) in each of the 3 days post dsRNA injection (*x*-axis). Vertical bars represent the mean with standard error (SEM) of 3 biological replicates. Each experiment consisted by minimum of 50 female sand flies in each experimental and control groups. Significant differences were calculated using two-way ANOVA with Holm-Sidak’s correction (*p < 0.05; **p < 0.01).

We hypothesized that defensins could have a role in the *L. major* cycle in the gut of *P. papatasi*. Therefore, we silenced them independently using 300 ng of gene-specific dsRNA, or concurrently using a mixture of 150 ng of each defensin dsRNA in infected sand flies. To assess the effect on *Leishmania* parasites we used sand flies treated with AtbC to eliminate the influence of the natural sand fly gut bacteria. We estimated the parasite abundance by the gene expression of the parasite actin in the dsDef1 and dsDef1+2. On day 6 post-infection, parasite numbers in both experimental groups were significantly increased compared to the control group inoculated by dsLacZ (Figure 4A).

Mortality was recorded during 3 days after dsRNA microinjections in infected sand flies to detect a possible negative effect of defensin silencing on *P. papatasi* survival. The mortality was slightly higher in both experimental groups during the entire course of experiments, but the most noticeable difference was observed during late-stage infections. On day 6, mortality reached roughly 15% in the control group, while in dsDef1 was more than 42%, and in dsDef1+2 was 34%. Statistical significance indicating a negative effect of silencing the defensin genes in infected flies was found in dsDef1 and dsDef1+2 injected group (Figure 4B).

**FIGURE 5:**
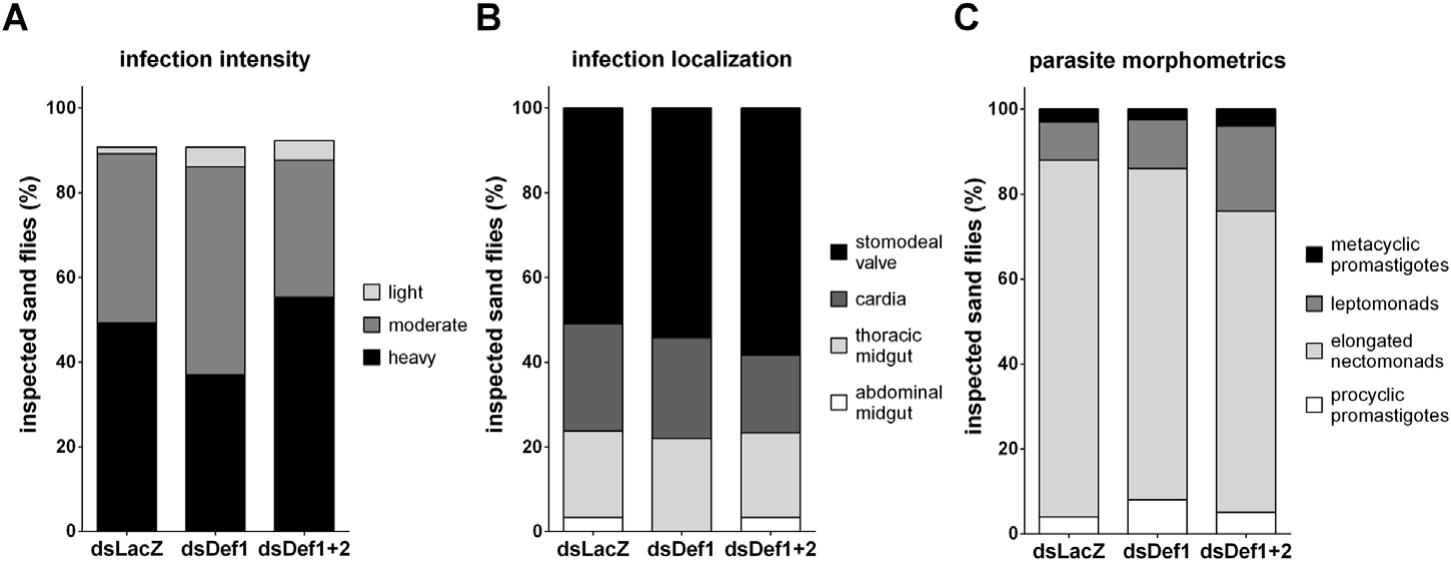
Effect of defensins silencing in the progress of *Leishmania* on the sixth day post-infection. **(A)** Infection intensity estimation with the *y*-axis representing the percentage of all individually inspected insects (minimum of 60 sand flies in each group). Bar colors indicate infection intensity. **(B)** Infection progress in the sand fly gut with the *y*-axis representing the percentage of infected insects. Bar colors indicate sand fly gut localization. **(C)** Parasite development in the sand fly gut with the *y*-axis representing the percentage of analyzed parasites. Bar colors indicate parasite developmental forms. The *x*-axis represents dsRNA-injected groups. Vertical bars represent the average values of three independent experiments. Significant differences were analyzed by Chi-square using both experimental and control groups. Fisher’s test was used between the experimental and control groups to assess the differences within each category.

In addition to the molecular detection of the parasite, we assessed the effect of the defensins gene silencing on the *Leishmania* infection load, parasite localization *in situ* and morphology by light microscopy. This method provides quick assessable information and it is applicable even to low-intensity infection after the blood digestion is completed (Myskova et al., 2008). A slight increase in moderate infections and a concomitant decrease in heavy infections in the dsDef1 injected group were not statistically significant (Figure 5A). A similar percentage of moderate and heavy infected sand flies were found between dsDef1+2 and the control group (Figure 5A).

In the two experimental and the control groups, parasites were able to colonize the stomodeal valve. In the control group, the stomodeal valve was infected in approximately 50% of examined infections in all groups (Figure 5B). Interestingly, in the control and dsDef1+2 groups, some of the infections remained localized only in abdominal gut, while in dsDef1, all inspected infections colonized also the thoracic parts of the gut (Figure 5B).

The parasite morphometric data analysis showed no significant difference among the procyclic and metacyclic promastigote forms between the dsDef1, dsDef1+2, and dsLacZ groups. Nevertheless, there was a significant (*p* = 0.0055) decrease in the percentage of the elongated nectomonads and a consequent increase in leptomonads in the dsDef1+2 sand flies compared to the control group (Figure 5C).

## 4 Discussion

Sand flies, like other insects, rely on innate immune mechanisms to fight potentially harmful microbial and viral challenges (Telleria et al., 2018). Nevertheless, it is not yet clear how the sand fly immunity affects *Leishmania* parasites. In this study, we focused mainly on two AMPs, a previously reported gut-specific defensin1 (Kykalová et al., 2021) and newly identified defensin2, their association with the relish transcription factor, and their role during *Leishmania* infection.

Based on phylogram analyses, the newly identified *P. papatasi* defensin2 (PpDef2) grouped with *L. longipalpis* defensin2 and *P. duboscqi* defensin. Moreover, *P. papatasi* defensin2 forms a wider clade with mosquito and other insect defensins. On the other hand, *P. papatasi* defensin1 (PpDef1) formed a group with *L. longipalpis* defensin4, which is enclosed in a clade with two other *L. longipalpis* defensins (Kykalová et al., 2021). These findings indicate a broad spectrum of insect defensins within the same sand fly species and their similarity across insect species.

We found that *PpDef2* gene was expressed in the gut and the rest of the body (carcasses), which includes the fat body. Nevertheless, a significantly higher expression was found in the carcasses which suggests a distribution of PpDef2 across various sand fly tissues. In contrast, the previously reported *PpDef1* gene was expressed exclusively in the *P. papatasi* gut and not detected in other tissues (Kykalová et al., 2021). Similarly, in *Anopheles gambiae*, the AMP gambicin was highly expressed in the anterior midgut while less expressed in other segments of the mosquito digestive tract (Vizioli et al., 2001). In *Rhodnius prolixus*, the defensin A, but not defensin B, was highly expressed in the fat body and less expressed in the anterior midgut when infected by *Trypanosoma cruzi* (Vieira et al., 2016). These findings indicate that some AMPs are expressed differently depending on the tissue or organ. The gut-specific PpDef1, or other AMP, may compensate for the low expression of PpDef2 in the gut. For example, *Drosophila* and *Tenebrio* insects used AMPs synergy to resist different pathogenic challenges (Zanchi et al., 2017; Hanson et al., 2019), but the such possibility was never investigated in sand flies.

During *Leishmania* infection, these two defensins showed a different expression pattern. While *PpDef2* did not show significant changes in the present study, *PpDef1* was upregulated on day 6 post-infection (Kykalová et al., 2021). These findings suggest that *PpDef1* and *PpDef2* genes were not concurrently expressed in the infected sand fly gut. These different defensins may be under the control of different transcription factors, therefore, under the control of different regulatory pathways. In *P. papatasi*, knockout of relish using CRISPR/Cas9 genome editing increased susceptibility to *Leishmania* infection (Louradour et al., 2019). However, the direct correlation between relish and the downstream expression of effector molecules in sand flies remained unknown.

We observed that the expression of *PpRel* gene was similar in both guts and carcasses samples, indicating that the IMD pathway can be regulated across *P. papatasi* body. Our results showed a significant reduction of *PpAtt* and *PpDef2* genes in the carcasses when *PpRel* was silenced with 150 ng of dsRNA, a commonly used amount of dsRNA in sand flies (Sant’Anna et al., 2008; Sant’Anna et al., 2009; Diaz-Albiter et al., 2011; Telleria et al., 2012; Telleria et al., 2021b; Telleria et al., 2021a; Di-Blasi et al., 2019). Nevertheless, *PpRel* silencing was not significantly reached in the guts with this same amount of dsRel. The subsequent *PpAtt* expression was quite variable, and defensins’ expression was observed with a slightly reduced expression of *PpDef2*. Our findings indicate that the *PpRel* silencing correlated to the downregulation of AMPs in carcasses, but this correlation remained unclear in the gut tissues. The correlation between relish knockdown and the suppressed AMPs production has been repeatedly reported in other insects such as *Galleria mellonella* (Sarvari et al., 2020) and *Octodonta nipae* (Sanda et al., 2019), indicating an evident conserved function. In *Tenebrio molitor*, the relish-silencing led to reduced levels of *attacin2* in the fat body and hemocytes but increased levels in the gut (Keshavarz et al., 2020). Therefore, in sand flies, the relish correlation with AMPs may also differ depending on the organs or tissues.

In addition to relish, we aimed to knock down the defensins genes in the adult sand flies. In the first set of experiments, we used 32.2 nL of 4.5 μg/μL dsRNA (150 ng). When injecting this initial amount of dsDef1 or dsDef2 in colony sand flies, we did not observe a reducing effect on the expression of the gut-specific *PpDef1* gene, while PpDef2 was significantly silenced in carcasses with the same dsRNA amount. This result indicated that 150 ng of dsRNA injected in *P. papatasi* was insufficient for obtaining a significant gene silencing in the gut tissue.

Consequently, to silence the defensins in sand fly guts during *Leishmania* experimental infections, we increased the amount of injected dsRNA to 64.4 nL of 4.5 μg/μL dsRNA (300 ng), and we obtained a significant silencing of *PpDef1* gene in guts. The additional strategy of injecting the same volume but composed of a mixture of two different dsRNA, 150 ng of each dsDef1 and dsDef2, was used for comparison purposes and resulted in the silencing of both defensin genes. These results suggest that a larger volume allowed a better distribution of injected dsRNA within the sand fly body, with the consequent silencing effect in the insect gut. Nevertheless, an opposite trend was previously reported when silencing another *P. papatasi* gene. A more efficient silencing was obtained with a lower concentration and volume (23 nL of 3.5 μg/μL) of a chitinase dsRNA (Coutinho-Abreu et al., 2010a). Together these findings support the hypotheses that different aspects such as dsRNA distribution, RNAi target region, or transcription turnover may be at play in successful gene knockdown (Pancoska et al., 2004; Dornseifer et al., 2015; Svoboda, 2020).

We hypothesized if defensins knockdown in infected females may influence sand fly mortality. In our experiments, the mortality rate on day 3 slightly increased in the dsLacZ control, while in both experimental groups (dsDef1 and dsDef1+2) increased sharply with significant differences. These findings are similar to defensins’ suppression in *A. gambiae* and *T. molitor* leading to the reduced viability of insects after G+, with a less remarkable effect after G-bacterial infection (Blandin et al., 2002; Zanchi et al., 2017). Therefore, the outcome of defensins’ activity varies with the pathogenic challenge.

Focusing on the possible effect of suppressed sand fly defensins on *Leishmania*, we investigated parasite levels based on the relative gene expression of a constitutive parasite gene in bacteria-depleted females. The depletion of bacteria in the infection experiments was chosen to reduce the additional effect of gut bacteria on immunity. The use of antibiotic treatment may have distinct effect on the parasite development. For example, antibiotic treatment alone had no deleterious effect on the parasites *Leishmania donovani* in *L. longipalpis* (Dey et al., 2018). On the other hand, reducing bacteria interfered negatively with *L. infantum* infection progress in the same sand fly, evidenced by the reduction of metacyclic promastigotes (Kelly et al., 2017). Under our experimental conditions, the choice of AtbC treatment reduced significantly the bacteria load in the sand fly gut and had no detectable effect on *L. major* development in *P. papatasi* (Kykalová et al., 2021). Therefore, it is plausible to consider that different factors such as choice of antibiotic combination, parasite, sand fly species and its commensal microbiota are influencing the effect of the antibiotic treatment.

Based on qRT-PCR results, we showed that the absence of defensins led to significantly increased levels of *Leishmania* in the insects silenced with PpDef1 or PpDef1+2 dsRNA. This trend was not observed in *Leishmania*-infected *L. longipalpis* females with *LlDef2* gene silenced (Telleria et al., 2021b). Nevertheless, several different factors must be considered. In our experiments, *P. papatasi* females were injected with dsRNA 3 days post *Leishmania* infection; therefore, the silencing effect lasted until 72 h post dsRNA injection corresponding to day 6 of infection, when the defecation process was finished. Differently, in the previous study done with *L. longipalpis,* the females were injected with dsRNA prior to the infection, with the silencing effect lasting until day 3 of infection when the *Leishmania* levels were measured by qPCR (Telleria et al., 2021b). Interestingly, the absence of attacin resulted in increased levels of *Trypanosoma* parasite in *Glossina morsitans morsitans* (Hu and Aksoy, 2006). Analogously, the overexpression of defensin A and cecropin A reduced number of *P. gallinaceum* oocyst in the infected *A. aegypti* females (Kokoza et al., 2010). On the other hand, in *A. gambiae* infected by *Plasmodium berghei*, the vector viability and the development of the parasite in the gut were not affected in the suppression of a defensin (Blandin et al., 2002). Altogether these observations highlight the differences that reflect the complex tunning of AMPs in regulating parasitic insect infection.

To help understanding the effects of defensins silencing in the development of the parasite inside the vector, we evaluated individually dissected gut using light microscopy. No statistically significant differences were observed in the localization of the parasite within the vector’s gut and infection intensity, despite the increased percentage of moderate infection in the dsDef1 silenced group. Similar negative results were previously observed in *L. longipalpis* infected by *L. infantum*: silencing of *LlDef2* gene did not result in significant differences in intensity, localization, and parasite morphology (Telleria et al., 2021b). Nevertheless, here in *P. papatasi* dsDef1 injected group, we observed that all insects had infections in the thoracic parts of the gut on day 6, suggesting that the parasites were developing faster than the other groups. Based on parasite morphology, we conclude that the absence of both defensins may provide room for the multiplication of the parasites when the non-multiplying elongated nectomonads forms were reduced at the expense of multiplying leptomonads.

It is possible that the PpDef2 gene silencing in the carcass had an influencing effect on the outcome of *Leishmania* infection in the gut. AMPs are readily induced in the gut but also in hemocytes after a potential risk, leading to other subsequent molecular signals that activate a broader systemic immune response (Krautz et al., 2014; Manniello et al., 2021). In addition, different pathway regulatory events may take place. For example, in *Drosophila*, the expression of the AMP drosomycin is regulated by the IMD pathway in response to tracheal epithelium infection. However, the Toll pathway regulates it during a systemic response (Ferrandon et al., 1998; Tzou et al., 2000). These findings revealed distinct regulatory mechanisms in insects for systemic and local induction of AMPs genes.

We emphasize that the gene silencing detected by qPCR indicates the decrease in mRNA levels, but not peptide levels. While complete suppression of mRNA and protein levels may not be obtained through RNAi-mediated gene silencing, significant differences between experimental and control groups can be attributed to the gene-specific mRNA suppression.

In summary, *Phlebotomus papatasi* has at least two defensin genes; one is gut-specific (*PpDef1*), and the newly investigated *PpDef2* is expressed throughout the sand fly tissues. The IMD pathway transcription factor relish regulated the expression of two investigated AMPs (PpAtt and PpDef2). We adjusted the dsRNA microinjections protocol and obtained successful gene silencing in the sand fly gut. The suppression of *PpDef1* or the combination of both defensins genes led to increased *L. major* loads and higher sand fly mortality. Moreover, we demonstrated the importance of defensins in the *P. papatasi* response toward *L. major*.

## Data Availability

The original contributions presented in the study are included in the article/Supplementary Material, further inquiries can be directed to the corresponding author.
