# Supplementary material for: RNAi-mediated gene silencing of Phlebotomus papatasi defensins favors Leishmania major infection
Source: Front Physiol. 2023 May 9;14:1182141. doi: 10.3389/fphys.2023.1182141 (PMC10230645; doi:10.3389/fphys.2023.1182141)
Supplement: Supplementary file 1 [file DataSheet1.docx]

Supplementary Material

RNAi-mediated gene silencing of *Phlebotomus papatasi* defensins favors *Leishmania major* infection

**Barbora Vomáčková Kykalová^1^, Fabiana Sassù^1^, Petr Volf^1^, and Erich Loza Telleria^1^***

*** Correspondence:** Erich Loza Telleria: erich.telleria@gmail.com

# Supplementary Figure 1


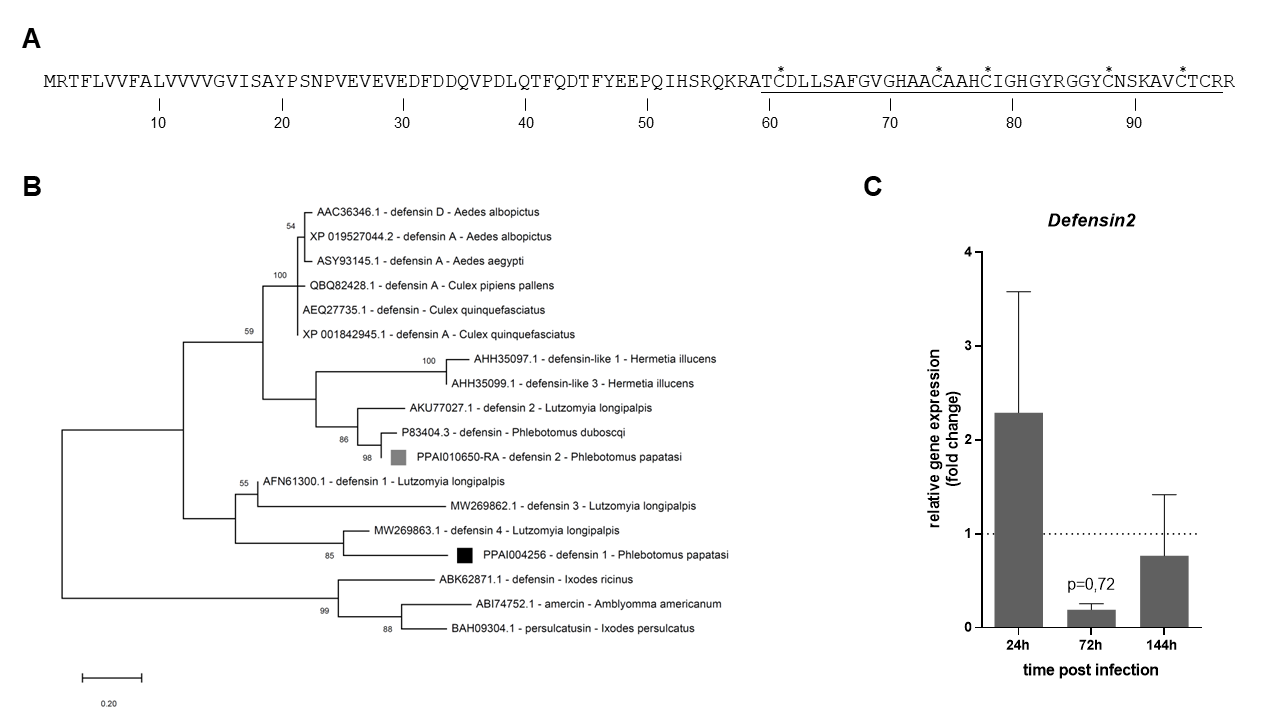


**Supplementary Figure 1. *Phlebotomus papatasi* defensins.** (A) PpDef2 predicted amino acid sequence (PPAI010650) with underlined defensin superfamily domain and highlighted conserved cysteines. (B) The phylogram of defensins was composed of amino acid sequences from *P. papatasi* and other arthropods, and the phylogenetic relationships were inferred by the Maximum Likelihood method with the WAG+I model. Tick AMPs sequences were used as an outgroup. Numbers on branch nodes indicate bootstrap values higher than 50 %. The *P. papatasi* defensin sequences are indicated by grey and black squares; species names are followed by corresponding Vector Base (*P. papatasi*) or GenBank (other species) accession numbers. (C) Expression of *PpDef2* gene in *P. papatasi* guts. The y-axis represents the relative expression normalized to endogenous control genes *PpAct* and *PpRibL8* and expressed as fold change compared to a non-infected blood-fed control group collected at each correspondent time point (dotted line). The x-axis indicates time points post-infection when samples were collected. Vertical bars represent the mean with standard error (SEM) of 3 biological replicates. No significant differences were found (Two-way ANOVA with Holm-Sidak's correction).

# Supplementary Figure 2

**Supplementary figure 2. Expression of *P. papatasi* defensin 2 gene in carcasses after dsRNA injection.** The relative expression of *PpDef2* gene in dsDef2-injected sand fly carcasses is represented in the y-axis, and time points when samples were collected post dsRNA injection are indicated in the x-axis. The relative expression was normalized to endogenous controls *PpAct* and *PpRibL8* genes and expressed as fold change compared to the dsLacZ control group collected at each correspondent time point (dotted line). Vertical bars represent the mean with standard error (SEM) of 3 biological replicates. Significant differences were calculated using two-way ANOVA with Holm-Sidak’s correction (**p< 0.01).

# Supplementary Figure 3





**Supplementary figure 3. Cross-check of *P. papatasi* defensins expression in gut samples of dsDef1 and dsDef2 injected sand flies.** (**A**) The relative expression of *PpDef1* gene in guts of dsDef2-injected sand flies and (**B**) *PpDef2* gene in guts of dsDef1-injected sand flies are represented in the y-axis. Time points when samples were collected post dsRNA injection are indicated in the x-axis. The relative expression was normalized to endogenous controls *PpAct* and *PpRibL8* genes and expressed as fold change compared to the dsLacZ control group collected at each correspondent time point (dotted line). Vertical bars represent the mean with standard error (SEM) of 3 biological replicates. No significant differences were found (two-way ANOVA with Holm-Sidak’s correction).
